# Rapid isolation of intact retinal astrocytes: a novel approach

**DOI:** 10.1186/s40478-023-01641-7

**Published:** 2023-09-25

**Authors:** Paul F. Cullen, Arpan G. Mazumder, Daniel Sun, John G. Flanagan

**Affiliations:** 1 Department of Ophthalmology, Schepens Eye Research Institute of Massachusetts Eye and Ear, Harvard Medical School, Boston, MA 02114 USA; 2 Herbert Wertheim School of Optometry and Vision Science, University of California at Berkeley, Berkeley, CA USA

**Keywords:** Retina, Retinal astrocytes, Glaucoma, Reactive astrocytes, Ribotag, Isolating astrocytes

## Abstract

Astrocytes are a major category of glial support cell in the central nervous system and play a variety of essential roles in both health and disease. As our understanding of the diverse functions of these cells improves, the extent of heterogeneity between astrocyte populations has emerged as a key area of research. Retinal astrocytes, which form the direct cellular environment of retinal ganglion cells somas and axons, undergo a reactive response in both human glaucoma and animal models of the disease, yet their contributions to its pathology and progression remain relatively unknown. This gap in knowledge is largely a function of inadequate isolation techniques, driven in part by the sparseness of these cells and their similarities with the more abundant retinal Müller cells. Here, we present a novel method of isolating retinal astrocytes and enriching their RNA, tested in both normal and ocular hypertensive mice, a common model of experimental glaucoma. Our approach combines a novel enzyme assisted microdissection of retinal astrocytes with selective ribosome immunoprecipitation using the Ribotag method. Our microdissection method is rapid and preserves astrocyte morphology, resulting in a brief post-mortem interval and minimizing loss of RNA from distal regions of these cells. Both microdissection and Ribotag immunoprecipitation require a minimum of specialized equipment or reagents, and by using them in conjunction we are able to achieve > 100-fold enrichment of astrocyte RNA.

## Introduction

Astrocytes are increasingly known to perform a variety of essential physiological roles in the central nervous system (CNS), including regulation of synaptic function, buffering ions and neurotransmitters, modulating immune response, blood brain barrier maintenance, contributing to neurovascular coupling, and provisioning energy to neurons [1, 2]. Additionally, under pathological conditions such as disease or injury, astrocytes undergo diverse morphological, transcriptional, and functional changes that can significantly influence outcome across a range of disorders [3]. These changes, collectively referred to as reactivity, are heterogeneous and highly context dependent—varying not only due to the nature and severity of the insult itself, but also in response to signals from neurons, other glia, and both resident and peripheral immune cells—and can include both supportive and detrimental alterations in astrocyte behavior [4, 5]. Alongside this improved understanding of the complex role of astrocytes in health and disease has come a newfound appreciation of their heterogeneity, both between and within CNS regions [6, 7]. The emerging variety of astrocyte subtypes and localized functions suggests that we cannot rely on a homogenous portrait of astrocyte behavior but must consider the intrinsic characteristics of astrocyte populations alongside their response to specific pathologies, particularly as we move towards treating these cells as potential druggable targets for effecting neuroprotection in the face of neurological injury and neurodegenerative disease.

Our research is focused on a small cluster of related astrocyte populations—those of the retina and optic nerve—that are thought to play a role in the progression of glaucoma, a widespread neurodegenerative disease characterized by the loss of retinal ganglion cells that is the leading cause of irreversible blindness [8–10]. While unique functions such as long-distance provisioning of energy substrates to neurons and enhanced phagocytic activity have been identified in astrocytes of the optic nerve and optic nerve head, less is known about retinal astrocytes [11–13]. Although they are known to play a critical role during developmental vascularization of the retina in many mammals, including humans, the study of retinal astrocytes is a particular challenge, and their functions in the adult retina are poorly understood [14]. The difficulty of studying these cells stems in part from their sparseness, as they make up approximately 0.1% of all retinal cells [15, 16]. While primary cell culture has historically been utilized to purify and expand retinal astrocyte populations, astrocytes are highly adaptive and rapidly alter their phenotype in vitro [17–19]. Moreover, the presence of Müller cells—specialized glia endogenous to the retina that outnumber retinal astrocytes by an order of magnitude, survive under similar culture conditions, and share many markers with astrocytes—further complicates in vitro investigation, and makes prospective isolation via cell sorting a significant challenge [20]. In situ assessment of these astrocytes—in live retina or fixed tissue—facilitates identification by preserving morphology, but limits investigation to highly targeted inquiries rather than the more unbiased approaches of modern ‘omics’-based investigation. Although several single cell RNA-seq studies of the retina have obtained a limited transcriptional profile from relatively small numbers of these astrocytes, these also have limitations [21–23]. Beyond well-known issues of ‘shallow’ sequencing depth, wherein only relatively high copy number transcripts are detected, astrocytes—like neurons—are known to engage in localized translation in their distal processes, which possess distinct RNA profiles linked to their interactions with vasculature and neuronal structures such as axons [24–26]. Loss of these elements during enzymatic dissociation for single cell sequencing can bias profiles of astrocyte gene expression and obscure pathways with physiological and pathological relevance.

In order to bypass these limitations, we developed a method specifically targeting retinal astrocytes.

Because they are located at the vitreal surface, we utilize a form of enzyme-assisted microdissection to dramatically enrich these astrocytes by mechanically separating them from the retina; we refer to this technique as a ‘pull-off’, in reference to similar early approaches to isolate retinal ganglion cells [27, 28]. This allows us to capture retinal astrocytes while excluding other cell types (including Müller cells); and has significant advantages in being rapid, requiring a minimum of specialized equipment and reagents, and preserving cell morphology for microscopic assessment. Our estimates of astrocyte enrichment resulting from this approach range from roughly one to two orders of magnitude. While the pull-off itself achieves significant enrichment of retinal astrocytes, we have been able to further enhance the signal from these cells in RNA-based and transcriptomic applications by incorporating subsequent immunoprecipitation of astrocytic RNA from pull-off samples via the well-established ‘Ribotag’ approach, which we have previously utilized to characterize the transcriptional profile of astrocytes of the optic nerve and optic nerve head [29, 30]. Immunoprecipitation results in enrichment of the astrocytic marker GFAP by an additional order of magnitude as measured by RT-qPCR, suggesting depletion of > 99% of non-astrocyte derived RNA, a result in excess of what either approach can achieve independently. We have further verified that this technique is also suitable for the isolation of astrocytes from retinas exposed to microbead-occlusion, an experimental ocular hypertension model that mimics key aspects of glaucoma.

## Detailed methods

The pull-off is a multi-step microdissection approach facilitated by enzymatic treatment. Initial dissection of the retina is followed by an incubation with collagenase to disrupt adhesion between retinal layers, after which a glass coverslip is gently lifted to ‘pull-off’ the innermost retinal layer for additional processing for applications such as immunostaining or RNA isolation, greatly enriching the signal from otherwise sparse retinal astrocytes. With the use of transgenic ‘Ribotag’ mice that express HA-tagged ribosomes in astrocytes (such as Ribotag x GFAP-Cre), mRNA from these cells can be purified further for applications such as RNA-seq and qPCR. These detailed methods describe the process from enucleation to immunoprecipitation (for pull-off plus immunoprecipitation), as well as an alternative stopping point for pull-off alone.

Immediately before dissection, prepare a working solution (75–150 U/ml, see Troubleshooting & Pitfalls) of collagenase and allow aPES filter to soak for 30 min in distilled H_2_0 before use. If continuing to IP or isolating RNA immediately after microdissection, prepare Homogenization Buffer with supplements before beginning and keep on ice during procedure; wash buffer should be prepared fresh with supplements on day 2. Asterisks (*) denote steps with specific troubleshooting recommendations featured in the Troubleshooting/Pitfalls section.

### Enzymatically assisted microdissection (‘Pull-off’)

**Fig. 1:**
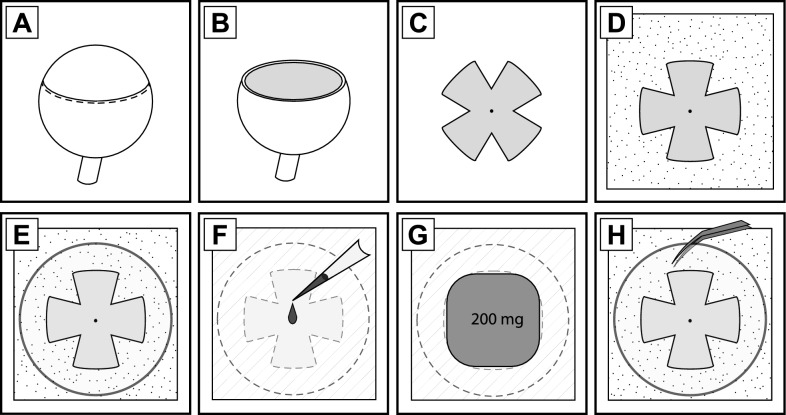
Diagrammatic representation of key steps in the ‘pull-off’. **A**: After enucleation of the eye, an incision is made immediately posterior to the limbus (as indicated by the dashed line) and the anterior segment is removed by cutting circumferentially. **B**: Afterwards, the lens and vitreous are removed, exposing the retina. **C**, **D**: Four relieving cuts are made in the retina, which is then dissected out and laid flat on aPES filter, with the photoreceptor side facing down and making contact with the matte surface of the filter. **E**: The retina and filter are placed with the retina facing up on blotting paper, and a glass coverslip is gently placed on the nerve fiber layer of the retina. **F**: The filter-retina-coverslip stack is carefully inverted, and 7 µl of collagenase working solution are added to the back of the filter. After this is absorbed, another 7 µl of collagenase working solution are added. **G**: Once the collagenase has been completely absorbed, a 200 mg weight is lowered onto the inverted stack. The blotting paper is used to lift the entire assembly and place it in a 100 mm petri dish, which is then covered and placed on a heating block at 37 °C to incubate. **H**: After incubation, the weight is removed and the stack carefully placed on dry blotting paper with the coverslip facing up. After brief additional drying, the coverslip is gently removed, separating the inner limiting membrane and retinal astrocytes from the underlying retina

1. After euthanizing the mouse, enucleate one or both eyes with blunt curved forceps, placing eyes in room temperature PBS*. Extraocular tissue, such as muscle, does not need to be removed from the eye and can in fact ease the dissection process. If both eyes are enucleated, the second can be kept at 4 °C to minimize postmortem changes while processing the first.
2. Transfer the first eye to a 35 mm petri dish with PBS; a small (1–2 cm^2^) piece of lab wipe can be submerged in the dish to act as a substrate and reduce movement of the eye during handling.
3. Using fine tipped forceps, grasp extraocular muscles or the conjunctiva to stabilize the eye with non-dominant hand. Using a #11 scalpel or fine-gauge needle, make a small puncture at the limbus, just posterior (< 1mm) to the edge of the cornea*.
4. Continue stabilizing tissue while using spring-scissors to cut circumlimbally around the eye, adjusting the position of the tissue in order to maintain as straight a cut as possible (Fig. 1a).
5. Once the anterior segment has been cut free of the eye cup, pull it away with forceps; the lens should come along with the removed tissue (Fig. 1b).
6. Excess vitreous inhibits adherence of tissue to the coverslip and its removal is essential. However, the presence of astrocytes at the retinal surface makes them vulnerable to rough handling. Although forceps or pipettes can aid in removing the vitreous, we primarily employ a fine-tip watercolor brush due to its flexibility and low risk of causing tissue damage*.
7. After removing vitreous, gently separate the retina from the choroid by sliding a blunt probe or forceps between the two, leaving the retina attached to the eye cup solely via the optic nerve.
8. Once the two surfaces are separated, push the eyecup down so the retina can be accessed from both vitreal and choroidal surfaces, without severing the optic nerve. Make a series of cuts from the edge of the retina towards the optic nerve, spaced at 90-degree intervals, with the cuts ending approximately ~ 500 µm from the optic nerve head.
9. Once all 4 cuts are made, ensure there is no remaining connection or vitreous at the periphery, remove lab wipe and excess debris from the dish, and sever the optic nerve so that the retina can float freely (Fig. 1c).
10. Carefully place pre-wetted aPES filter (see ‘Preparation of Reagents’) matte side up in the petri dish, ensuring that the retina does not become pre-emptively stuck to the filter.
11. Lower the retina photoreceptor side down onto the filter by carefully removing PBS from the dish until the retina adheres in the center of the filter. Use the brush or forceps to control the orientation of the tissue and ensure that each of the four ‘lobes’ or ‘petals’ of the tissue lie flat with the nerve fiber layer oriented upwards (Fig. 1d).
12. With the retina flat on the filter, remove any remaining PBS and lift the filter.
  It is likely that additional liquid must be removed, but the sample should not be dried excessively. Briefly placing the filter on the back of a gloved hand (with the gloss side of the filter, opposite to the retina, contacting the glove) can draw away moisture without the filter sticking or the retina drying out. Ideally, the filter will remain wet but any visible liquid on the surface should be rapidly wicked away.
13. With the retina facing upwards, place the filter on the blotting paper, and gently lower a clean glass coverslip onto the retina, centering it as well as possible to form the glass-retina-filter stack (Fig. 1e). After ~ 10 s, grasp aPES filter with forceps and invert the stack; if enough vitreous has been removed, the glass will stick to the tissue. Place the stack glass-side down on the blotting paper.
14. Administer 7 µl of collagenase working solution to filter directly over the tissue; depending on how much the sample and filter have dried this should be absorbed in 10 s to a minute. Once the last of the solution disappears from the surface, add another 7 µl, which may absorb slightly more slowly (Fig. 1f).
15. Transfer the stack and blotting paper to a 100 mm petri dish and place a ~ 200mg weight directly over the tissue (Fig. 1g). Close the lid of the dish and incubate at 37 °C for 23 min on a heat block*.
16. After incubation, open the petri dish and remove the weight from the stack. Return the stack to the dissection scope, and gently invert so that it is once more glass side up.
17. Wait an additional minute to allow for final drying/adherence, then use angled or curved fine tip forceps to slowly work around underneath the edge of the glass and begin levering the tissue off. The inner limiting membrane and cells of the nerve fiber and retinal ganglion cell layers should visibly adhere to the glass, giving it a “frosted” appearance* (Fig. 1h).
18. Once the tissue is isolated, it can be treated with fixative for immunohistochemistry (IHC) purposes or appropriate buffer for RNA handling (RNA buffer for direct RNA isolation, or homogenization buffer for immunoprecipitation). In either case, we typically transfer the coverslip (tissue side up) to a well on a 24 well cell culture plate. This simplifies further handling and treatment, while reducing the volume of reagents needed per sample.
  For IHC, we utilize 10–15 min fixation at room temperature with 4% PFA; due to the thinness of the sample it can subsequently be stained with protocols suitable for thin sections or cell culture. Care should be taken with wash steps as the isolated tissue is delicate, and excess drying during this process can impact antibody performance.

### Immunoprecipitation of RNA

This section details our approach for homogenizing the tissue and performing immunoprecipitation to further enrich astrocyte specific RNA from Ribotag tissue. Ribotag mice express a modified version of the 60S ribosomal gene Rpl22 under the control of a Cre driver; by regulating Cre recombinase expression with a cell type specific promoter, the population of interest can be induced to express modified Rpl22 ‘tagged’ with a hemagglutinin (HA) epitope. This enables the isolation of tagged ribosomes from cells of interest via immunoprecipitation using an HA antibody, and the purification of associated mRNA for downstream analysis. In this case, we utilize a GFAP-Cre line we have previously employed to study optic nerve astrocytes; while the tendency of Müller cells to express GFAP in mouse models of ocular hypertension such as microbead occlusion would normally impair the viability of this approach, they are largely absent from samples obtained by pull-off. The advantages of this approach are highly complementary with the pull-off method: by isolating ribosomes and associated RNA directly, the need for a single cell suspension and prospective sorting is elided, reducing transcriptional changes that can result from extended post-mortem intervals and retaining RNA from distally expressed genes.

To isolate RNA directly without additional enrichment, perform homogenization (steps 19–27) with RNA isolation buffer, such as Qiagen Buffer RLT supplemented with β-mercaptoethanol, then proceed to purify RNA per your protocol’s instructions. Immunoprecipitation entails a 4-h primary incubation and a secondary incubation performed overnight (12–16 h). Samples can be kept frozen prior to homogenization.

19. After collecting isolated tissue on a coverslip, it can be kept on ice while other samples are isolated. Longer storage at − 20° or − 80 °C is possible, but caution should be used to avoid thawing and refreezing, which can occur quite rapidly due to the thinness of the tissue. DO NOT add homogenization buffer if freezing the sample.
  If samples are processed fresh, rather than frozen, it may be simpler to utilize the lid of the multiwell plate for sample holding, as this makes for easier handling during the following steps relative to the bottom of the well.
20. Place the multi-well plate with fresh or frozen samples on ice. If samples have been frozen, allow to equilibrate before adding homogenization buffer, as extreme cold can trigger freezing of the buffer and impact the quality and yield of RNA isolation.
21. Center the coverslip in the well and add 50 µl of Homogenization Buffer to each sample, placing it as a droplet directly over the tissue. Ideally, the tissue is near the center of the coverslip and surface tension will keep the droplet intact, minimizing sample loss*.
22. Homogenization Buffer should be allowed to sit on ice for at least 1 min, and up to 10 min if multiple samples are being processed at once. After, mechanically homogenize the tissue by ‘scraping’ with a 200 µl pipette tip, held like a pencil rather than attached to a pipette. This should be done firmly but cautiously, ensuring that the tip scrapes against the entire surface area of the sample while taking care not to spill the liquid off the coverslip.
  Should liquid spill off the coverslip, carefully aspirate it and transfer to the collection tube. Maximizing the recovery of homogenate will minimize the impact of spillage on RNA yield.
23. Attach the tip used for homogenization to a pipette and transfer the homogenate to a 1.5 ml collection tube, also on ice. Briefly triturate the mixture. Fragments of sample may remain visible and act as an indicator that the transfer was successful. Change tips and add 50 µl of homogenization buffer to the coverslip to prevent drying.
  Using the same tip for scraping and collection minimizes sample loss.
24. Proceed to homogenize and collect any additional samples, each in their own collection tube, adding 50 µl of fresh Homogenization Buffer to the coverslip after each.
25. After collecting homogenate from each sample, repeat the scraping and collecting process, adding the second batch of homogenate to the initial mixture from that sample. Triturate once more and add another 50 µl of buffer to each coverslip after collection.
  A brightfield inverted microscope, of the type used to inspect cell culture plates, can be used to check the coverslips for residual tissue.
26. Repeat the homogenization, collection and trituration step a third and final time; once this is completed vortex the samples for 30 s to 1 min in 15 s intervals, returning the tubes to ice briefly between vortexing to make sure they remain cold.
27. Centrifuge samples at 10,000 rpm for 10 min in a pre-cooled centrifuge at 4°C. Collect the supernatant and transfer to a new pre-cooled tube for each sample.
  A pellet may or may not be visible at the bottom of the centrifuged tube. A visible pellet is generally a reliable sign that the process thus far has worked, but absence of a visible pellet does not preclude a successful isolation.
28. A sub-sample of supernatant can be taken to serve as an input sample, which provides a useful reference for assessing immunoprecipitation efficacy via qPCR. To do so, take 10–20 µl of the supernatant and add it to 350 µl of RNA buffer, which can then be purified during the 4-h incubation of the immunoprecipitation sample or frozen at − 80 °C for long term storage*.
29. Add 2 µl each of anti-HA antibody to the remaining supernatant from each sample and incubate for 4 h at 4 °C on the rotator.
30. Approximately 30 min before the end of the primary incubation, equilibrate magnetic beads. Gently resuspend beads and transfer 50 µl of bead suspension per sample to new 1.5 ml tubes, then place in magnetic rack on ice for ~ 1 min to settle.
31. After beads settle, remove the buffer while keeping tubes in magnetic rack. Add 150 µl of Homogenization Buffer to each tube, then place on rotator for 30 min at 4 °C.
32. When the primary incubation and bead equilibration are complete, transfer tubes with beads to magnetic rack on ice and allow to settle for 1 min. Remove the Homogenization Buffer used for equilibration and transfer the incubated lysate/antibody sample suspension to the corresponding bead tube. Gently mix by pipetting and repeat for each additional sample, before returning them to the rotator for overnight incubation at 4 °C.

The following steps occur on day 2 of the immunoprecipitation. Afterwards, RNA can be purified via a commercially available kit (we use the RNeasy Micro Plus from Qiagen) and quantified (we use the Agilent Pico kit) before cDNA library preparation or other downstream applications.

33. Prepare Wash Buffer with supplements.
34. Remove tubes from the rack after overnight incubation, briefly spin down at low speed to ensure sample is not trapped in the lid of the tube. Place tubes in the magnetic rack on ice and allow beads to settle for at least 1 min.
35. Remove supernatant. This contains the unbound lysate fraction not conjugated to the antibody-magnet complex and can typically be disposed of but can also be kept at − 80 °C for troubleshooting.
36. Add 150 µl of Wash Buffer to each tube, remove from the rack and mix by gently pipetting. Return to the rack and allow tubes to settle for at least 1 min, then remove buffer. Repeat two more times for each sample.
37. To break linkage between beads and ribosomes (and the associated RNA that is the target of this approach), after the final wash with Wash Buffer add 350 µl of RNA Buffer to the tube. Vortex 30 s, then return samples to the magnetic rack on ice.
38. After allowing samples to settle for at least 1 min, collect supernatant and store at -80°C or proceed to RNA purification*.

## Additional methods

### Animals

All animals were handled in accordance with the ARVO Statement for the Use of Animals in Ophthalmic and Vision Research, and all procedures were approved by the Institutional Animal Care and Use Committee at Schepens Eye Research Institute. Mice aged 3–4 months were housed in a 12 h light/dark cycle and received food and water ad libitum. Two mouse strains were used in this study: (1) the Ribotag strain, B6J.129(Cg)-Rpl22tm1.1Psam/SjJ (The Jackson Laboratory, Bar Harbor, ME, strain #029977), and (2) B6.Cg-Tg (GFAP-Cre) 73.12Mvs/J (The Jackson Laboratory, Bar Harbor, ME, strain #012886). Further details of the Ribotag mouse can be found in the main body of the text. Ribotag mice can be crossed to a variety of Cre recombinase driver mouse lines, and in our case, we use a GFAP-Cre line in which a mouse GFAP promoter sequence directs expression of Cre recombinase in astrocytes. Both mice lines were on a C57Bl/6 background. We initially crossed GFAP-Cre recombinase expressing mice with hemizygous Ribotag mice to get offspring heterozygous for the Ribotag allele (Rpl22^HA/+^). These heterozygous Ribotag mice were then bred together to obtain experimental mice that were homozygous for the Ribotag allele with the Cre transgene (Rpl22^HA/HA^ Cre +).

### Microbead model

The microbead occlusion model was used to elevate intraocular pressure (IOP) unilaterally. Microbeads are inert and have been used in numerous studies of experimental glaucoma [14, 17, 30]. A glass microneedle was introduced through a corneal puncture created by a 30.5-gauge needle and 2.5 µl of 15 µm diameter polystyrene microbeads (Thermo Fisher Scientific; #F8841) were injected (final concentration of 2.7 × 10^7^ beads/ml suspended in saline). This method induces an elevated IOP that lasts 4 weeks and peaks at 23–27 mmHg at 7–10 days post-injection. IOPs were measured in both eyes 1 d before microbead injection and then every 3 d afterward using a tonometer (TONOLAB; Icare). As this was a method study, mice were sacrificed on day 7. The contralateral eye was untreated. Mice were regularly examined on a slit lamp for signs of any inflammatory response or overt damage in the anterior segment. Mice that showed any of these signs were excluded.

### Immunohistochemistry and astrocyte quantification

Isolated pull-off specimens were fixed 10–15 min with 4% paraformaldehyde in 1 × PBS at room temperature. Samples were washed 3 × with PBS, then incubated with blocking buffer (10% donkey serum, 0.5% Triton-X, 1% BSA in 1 × PBS) for 1 h. Following blocking, samples were incubated with primary antibodies for 1 h at room temperature, then washed 3 × with PBS and incubated with secondary antibodies for 1 h at room temperature. Afterwards samples were counterstained with Hoechst 33,342 (1 µM) for 15 min, washed three additional times with PBS, and mounted on slides with Prolong Diamond Antifade Mountant (Thermo Fisher Scientific). Imaging was performed on a Leica TCS SP8 confocal microscope. For quantification, 4 images per sample were taken midway between the retinal periphery and optic nerve head, with manual counting of astrocytes based on GFAP expression and total cell numbers determined by Hoechst-stained nuclei. Reference values for retinal astrocyte density and overall density of retinal cells were taken from previously published studies and used in calculations of enrichment [15, 16].

### RNA-purification, assessment, and cDNA library generation

After resuspension in RNA buffer (Qiagen Buffer RLT + β-mercaptoethanol), total RNA was purified via Qiagen RNeasy Plus Micro Kit per the kit’s instructions for microdissected samples and eluted in 10–12 µl of nuclease-free water. 1 µl of elute from each sample was analyzed using the Agilent 2100 bioanalyzer with the Agilent RNA 6000 Pico kit to quantify total RNA yield and RNA integrity (RIN). 500 pg of each sample was then used to generate cDNA via the Takara Smart-seq HT Kit.

### RT-qPCR

Quantitative PCR was performed via the Applied Biosystems StepOnePlus Real-time PCR system using Sybr Green PCR Master Mix as described previously [30]. Glyceraldehyde phosphate dehydrogenase (GAPDH) was used as a reference/housekeeping gene, and technical replicates (3x) were performed for each gene of interest from each sample. The ddCT method was used to normalize all gene expression to GAPDH.

## Results

### Assessment of pull-off alone and combined pull-off with immunoprecipitation in naïve retinas

**Fig. 2:**
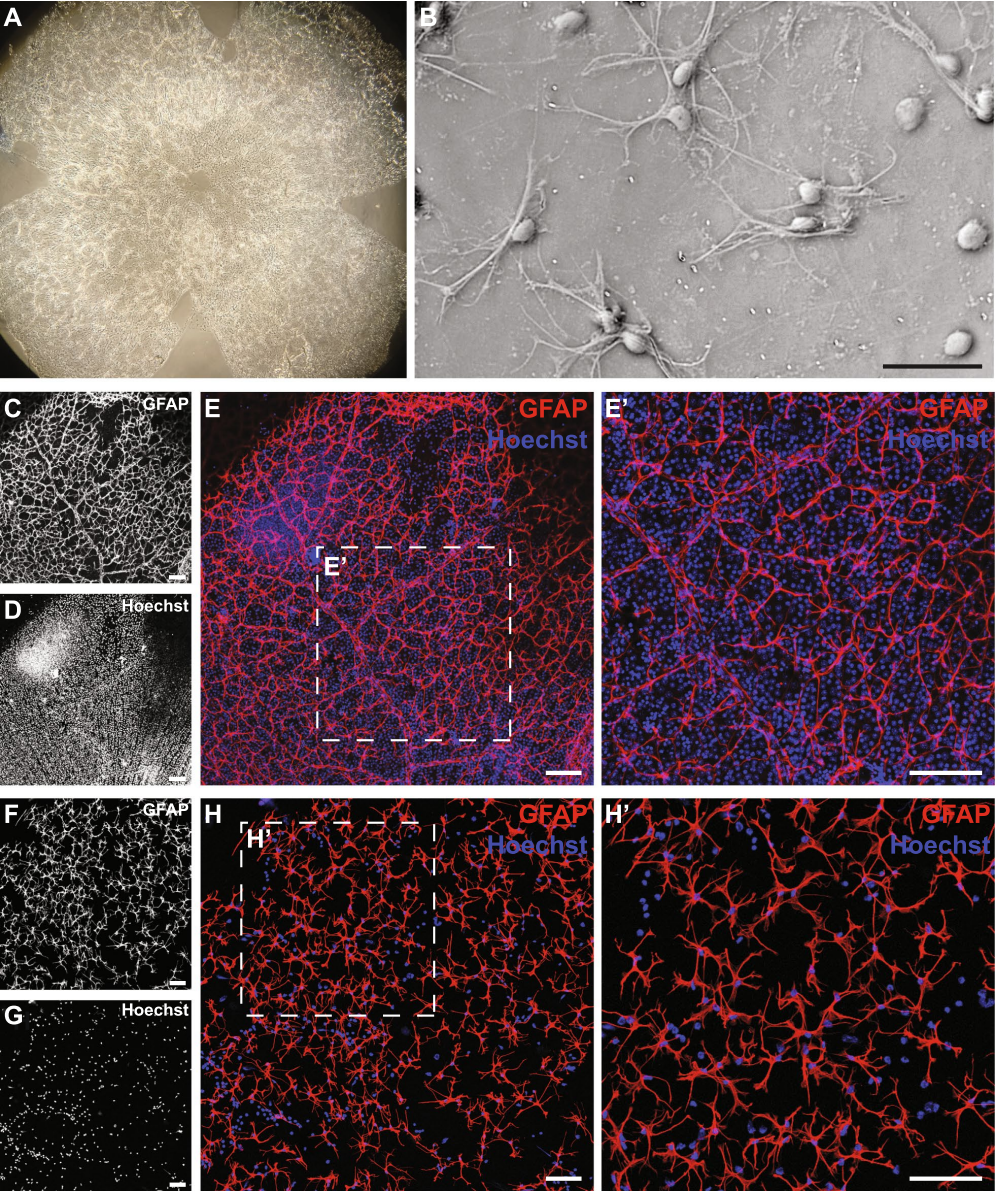
Representative images of isolated retinal astrocytes after pull-off. **A**: The pull-off captures astrocytes from across the vitreoretinal surface, forming a thin visible layer on the coverslip, viewed here through a dissection microscope. **B**: Pull-off preserves much of the morphology of retinal astrocytes as can be seen in brightfield with phase contrast. Scale bar = 50 µm. **C–E**′: Images of whole mounted retina showing astrocytes (**C**) and overall cell density (**D**) in the nerve fiber and retinal ganglion cell layers of the intact retina before pull-off. Merged Hoechst and GFAP immunostaining shown in E, with a higher magnification inset (**E**′). Scale bar in all panels = 100 µm. **F**–**H**′: Pull-off results in large scale isolation of astrocytes (**F**) and major reductions in the number of other cells (**G**), with combined immunostaining shown in (**H**) and a higher magnification inset (**H**′). Scale bar in all panels = 100 µm

To evaluate the pull-off during initial process development we relied on qualitative immunostaining and microscopy to verify the isolation of retinal astrocytes, an approach facilitated by the histological preservation that we consider a major advantage of this microdissection method. Preliminary assessment of pull-off isolated specimens via direct brightfield observation (Figs. 2a, b) revealed widespread adhesion of morphologically intact and recognizable astrocytes. Immunohistochemical staining for the astrocytic marker GFAP confirmed the identity of these astrocytes, which are isolated in abundance comparable to whole mounted retinas (Figs. 2c, f), while nuclear staining with Hoechst conversely reveals the mass depletion of GFAP^−^ cells (Figs. 2d, g).

Next, we employed RNA-based approaches to assess the efficacy of the pull-off, both alone and in conjunction with immunoprecipitation, and measure enrichment. Although relative, RT-qPCR has the advantage of enabling direct comparison of the pull-off with and without immunoprecipitation. It also provides a highly representative assessment of the method’s utility for RNA-based transcriptomic studies, its primary intended application. As this method was developed to facilitate transcriptional analysis of retinal astrocytes under a variety of conditions, we characterized yield, integrity, and purity of isolated RNA from untreated mouse eyes as well as those exposed to elevated IOP induced by microbead occlusion.

To assess RNA yield and integrity, we utilized the Agilent 2100 Bioanalyzer with the RNA 6000 Pico kit, which is suitable for RNA samples from micro-dissected tissue (50 pg -5 ng total RNA). After homogenization, we extracted RNA directly from a pull-off only input fraction consisting of 10% of the lysate, while the remaining 90% underwent subsequent immunoprecipitation prior to RNA extraction. Analysis of pull-off samples (*n* = 4) gave an average of 2.2 ng RNA (1–3.4 ng) and an average RIN value of 8.6 (7.8–9), indicating the full amount of RNA in microdissected tissue from a mouse retina is approximately 22 ng. After subsequent immunoprecipitation, samples (*n* = 4) averaged yields of 1.9 ng RNA (0.8–2.7 ng) with RINs of 8.8 (8.1–9.9), indicating that the immunoprecipitated RNA is approximately 8.6% of the roughly 22 ng of RNA present in microdissected tissue.

Having established the isolation of usable quantities of good quality RNA by both pull-off alone and with immunoprecipitation, we next sought to quantify enrichment of astrocytic RNA with 2-step RT-qPCR to assess enrichment or depletion of markers of astrocytes (GFAP), Müller glia (Cralbp), and retinal neurons (NeuroD1, Pax6), with the housekeeping gene GAPDH as control [31, 32]. For this application, we also generated whole retina control (WRC) samples by briefly homogenizing mouse retinas via sonication, centrifuging the lysate to remove debris (as above), and diluting a suitable volume of supernatant (~ 10 ul) in 350ul RNA buffer for purification. Purified RNA from pull-off, pull-off plus immunoprecipitation, and WRC samples were assessed for yield and integrity, as above, and cDNA libraries were generated using the Takeda Smart-Seq HT Kit with 500 pg of purified total RNA as template.

**Fig. 3:**
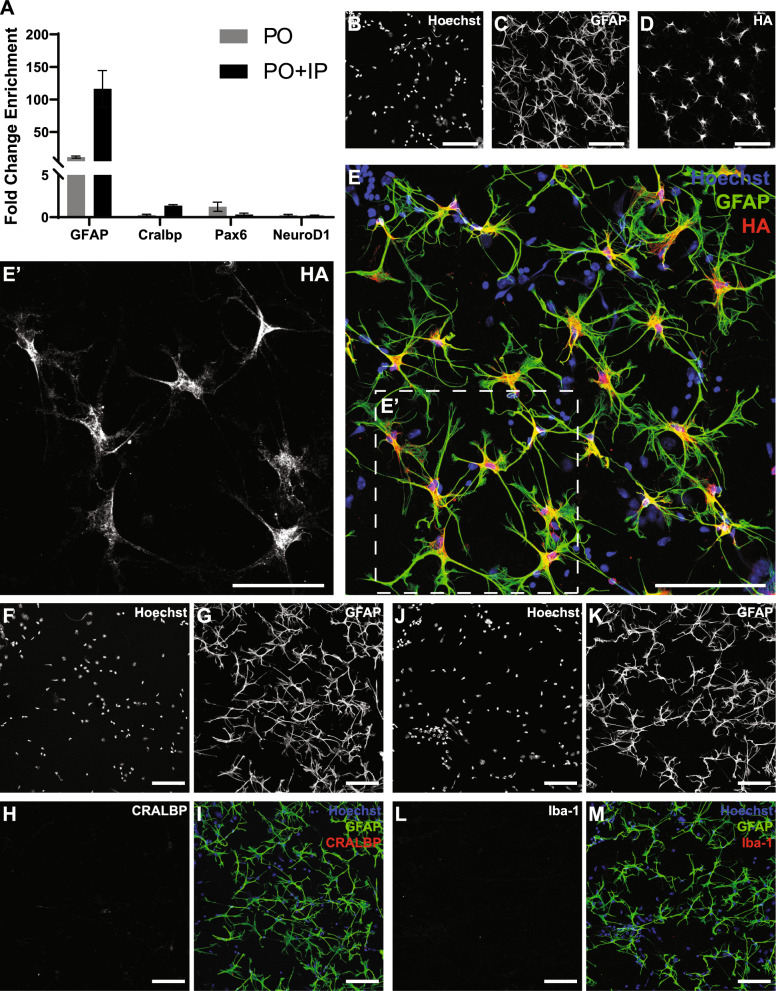
RT-qPCR and immunohistochemistry of astrocytic enrichment from pull-off and pull-off plus immunoprecipitation in naïve mouse. **A**: RT-qPCR results showing fold-change enrichment of markers of astrocytes (GFAP), Müller cells (Cralbp), inner retinal neurons (Pax6), and outer retinal neurons (NeuroD1) in pull-off (PO) and pull-off plus immunoprecipitation (PO + IP), relative to whole retina control. **B**–**E**′: Immunostaining of pull-off isolated tissue shows colocalization of astrocytes (**C**) with HA (**D**). Although few non-astrocyte cells remain after the pull-off (**B**), the restriction of HA-tagged ribosomes to astrocytes enables further enrichment of astrocytic RNA during the ensuing immunoprecipitation. HA-tagged ribosomes are present throughout the processes of isolated astrocytes (**E**′). Scale bar in panels **B**–**E** = 100 µm and in **E**′ = 50 µm. **F–I**: Absence of Iba-1^+^ microglia on pull-off isolated tissue. Scale bar in all panels = 100 µm. **J**–**M**: Absence of Cralbp^+^ Müller cells on pull-off isolated tissue. Scale bar in all panels = 100 µm

Relative to RNA from WRC samples, the astrocytic marker GFAP was enriched by a mean of 11.4-fold after pull-off (*n* = 6) (Fig. 3a), consistent with our visual assessment of microdissected samples via immunofluorescence and brightfield microscopy revealing non-astrocytic GFAP^−^ cells to be only sparsely adherent (Fig. 2c–h). The addition of immunoprecipitation improved overall enrichment still further to 116.2-fold (*n* = 6); this roughly tenfold enrichment from pull-off alone to pull-off plus immunoprecipitation comports well with the depletion of approximately 90% of input RNA by immunoprecipitation, indicating that this process is highly selective for HA-tagged ribosomes from GFAP^+^ astrocytes. Further corroborating this conclusion, co-staining of GFAP and HA on pull-off isolated tissue shows clear localization of HA to GFAP^+^ astrocytes (Fig. 3b–e) and confirms the presence of tagged ribosomes in astrocytic processes (Fig. 3e′).

With regards to markers of non-astrocytes, RT-qPCR results (Fig. 3a) showed NeuroD1—found predominantly in photoreceptors and interneurons in the outer retina—to be depleted by 77% (*n* = 5) by pull-off alone, with a total reduction in signal of 88% (*n* = 5) achieved by pull-off with immunoprecipitation. The inner retinal marker Pax6 also showed depletion in pull-off plus immunoprecipitation samples, with a net reduction of 68% (*n* = 5) vs WRC. Finally, the Müller cell marker Cralbp/Rlbp1 decreased by 75% (*n* = 4) in pull-off samples and was not detected by immunostaining of microdissected samples (Fig. 3F–I), indicating their selective removal. We also initially sought to use RT-qPCR to verify the depletion of microglia via the marker Iba-1; however, microglia in the retina are roughly as sparse as astrocytes and perhaps as a consequence, our results were inconclusive (data not shown) [33]. Immunostaining for Iba-1 on pull-off samples did not detect microglia, however, suggesting significant depletion or elimination by the pull-off itself (Figs. 3j–m).

Lastly, we employed immunostaining and quantitative microscopy to further assess the purity of astrocytes isolated by the pull-off. Although RT-qPCR was our preferred assay for the reasons described above, due to its relative nature it provides only limited information about the extent of astrocyte enrichment. We performed additional imaging to quantify enrichment of astrocytes by pull-off, manually counting total cells and GFAP^+^ astrocytes in regions midway between the optic nerve head and retinal periphery. Our quantitative assessment of these images identified an average of 60 ± 4 (SEM; *n* = 4) astrocytes per field of view, out of 245 ± 42 (SEM; *n* = 4) total cells, indicating that retinal astrocytes comprised approximately 24.5% of isolated cells. Given that astrocytes constitute approximately 0.1% of cells in the intact retinal, this metric suggests substantially higher enrichment than found by RT-qPCR, a finding we address in greater detail in the discussion section.

### Validation of enrichment from microbead treated tissue

Having established the efficacy of the combined pull-off plus immunoprecipitation approach on naïve retina, we next sought to confirm its suitability to study transcriptional changes resulting from microbead-induced ocular hypertension. As the reactivity induced in Müller cells by elevated IOP includes upregulation of GFAP, we were initially concerned that the selectivity of the method might be diminished under these conditions despite prior evidence of their depletion. To address this concern, we collected retinal tissue from microbead-treated animals (MB-tx) (*n* = 3) after 7 days of elevated IOP and isolated pull-off and pull-off plus immunoprecipitation RNA as with untreated animals. Microbead-treated WRC RNA was collected as previously described to act as controls, as were a limited number of additional pull-off samples from MB-tx retinas for immunohistochemical analysis.

**Fig. 4:**
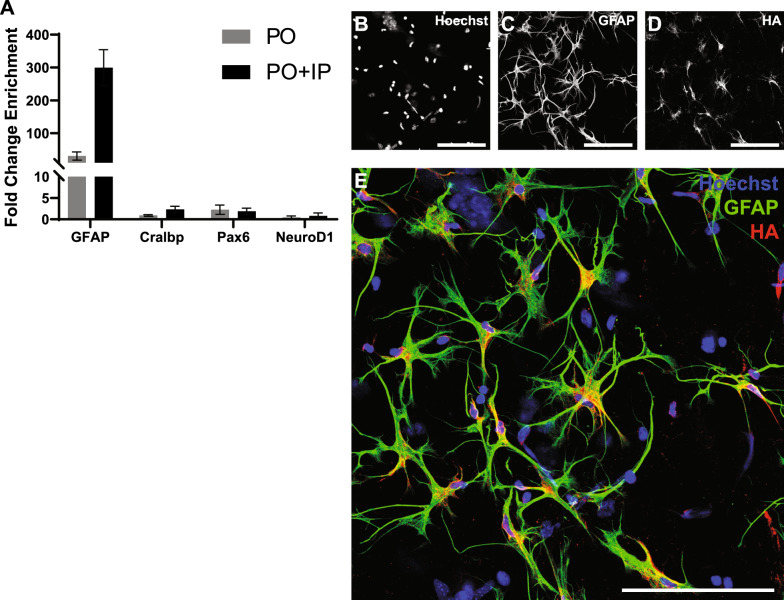
RT-qPCR and immunohistochemistry of astrocytic enrichment from pull-off and pull-off plus immunoprecipitation in microbead-injected mice. **A**: RT-qPCR results from microbead-treated animals showing fold-change enrichment of markers of astrocytes (GFAP), Müller cells (Cralbp), inner retinal neurons (Pax6), and outer retinal neurons (NeuroD1) in pull-off (PO) and pull-off plus immunoprecipitation (PO + IP), relative to whole retina control. **B**–**E**: Immunostaining of pull-off isolated tissue from microbead injected mice. Microbead treatment did not interfere with the isolation process, and HA continues to colocalize with astrocytes as in naïve mice. Scale bar in all panels = 100 µm

Strikingly, enrichment of GFAP mRNA by both pull-off and pull-off plus immunoprecipitation was retained and even enhanced. Comparison with MB-tx WRCs showed mean increases of 30.6-fold for pull-off alone and 300-fold after subsequent immunoprecipitation (Fig. 4a). Consistent with our RNA-based analysis, immunostaining of MB-tx pull-off samples demonstrated continued colocalization of GFAP and HA-tagged ribosomes in astrocytes (Fig. 4b–d). As with tissue from untreated animals, the large gap between GFAP enrichment and levels of neuronal (Pax6, NeuroD1) and Müller cell (Cralbp) markers persisted (Fig. 4a). Although only NeuroD1 continued to show outright depletion (56% for pull-off, 18% for pull-off plus immunoprecipitation), Pax6 and Cralbp levels remained near WRC baseline in both pull-off and pull-off plus immunoprecipitation samples after MB-tx, in strong contrast to the two orders of magnitude enrichment of GFAP. Although quantitative microscopy was not performed on microbead-treated samples, the appearance of these samples (Fig. 4e) did not differ qualitatively from those from untreated retinas (Fig. 3e).

## Discussion

Our results show clear isolation of retinal astrocytes and strong, selective enrichment of astrocytic RNA, as quantified by GFAP mRNA, from both naïve mouse retinas and those exposed to experimentally elevated IOP. The incorporation of subsequent purification via Ribotag immunoprecipitation enhances enrichment still further, with RT-qPCR results indicating > 100-fold enrichment, allowing for clear separation of markers of astrocytes and non-astrocytes. The combination of these two techniques exceeds what either approach is able to achieve independently, with the initial pull-off depleting highly abundant outer retinal neurons and Müller cells, and the subsequent immunoprecipitation facilitating the depletion of RNA from Pax6^+^ cells of the inner retina and ganglion cell layer. The merits of the combined approach are particularly pronounced in the case of the microbead model, as the presence of GFAP-reactive Müller cells would potentially confound attempts to purify astrocytic RNA by Ribotag immunoprecipitation alone.

Astrocytes of the retina have been regarded as a distinct population of glial cells for decades and appear to play major roles both in retinal development and disease-driven loss of retinal ganglion cells, but much of their behavior remains unknown [20]. Retinal astrocytes share key characteristics with white matter astrocytes, including fibrous morphology and close association with axons, yet populate an unmyelinated region, an unusual trait that they share with optic nerve head astrocytes [9, 34]. Early work in astrocyte calcium imaging and dye coupling in rodent retinas demonstrated the presence of a complex asymmetrical network coupled by gap junctions, while more recent work suggests that these networks may be involved in the spread of energy substrates across anatomically large distance [11, 35, 36]. Other studies have indicated heterogeneity within retinal astrocytes, both morphologically and with regards to expression of secreted factors. Computational analysis found that vein-associated astrocytes are larger than those associated with arteries, while an investigation of angiopoietin-4 (a ligand for the endothelial receptor Tie2) showed that it is selectively expressed in vein-associated astrocytes in the retina—especially the outer retina—but not found in other astrocyte populations [37, 38]. Finally, a very recent study in the retina highlights variation in size and morphology of these astrocytes, while reaffirming their extensive association with both retinal ganglion cells and the vasculature [39].

The largely descriptive nature of retinal astrocyte research hints at the current limitations of our ability to investigate these cells, and existing techniques for cell isolation or cell-type-specific RNA enrichment, including approaches that have been previously validated on astrocytes of the brain, are generally unsuitable for resolving pertinent questions about their function. Here we have demonstrated a technique, influenced by prior work on retinal ganglion cells, that exploits the localization of these astrocytes to circumvent the issue of their sparseness and facilitate their enrichment. This technique, an enzymatically-assisted microdissection, requires little in the way of specialized equipment or reagents and can be performed rapidly (< 1 h) and without disruptive dissociation of tissue to a single-cell suspension, minimizing post-mortem alterations to gene expression or loss of signal from genes with polarized expression.

While there is divergence in our quantification of enrichment by immunohistochemistry and RT-qPCR from pull-off only samples, we suspect this reflects differences in sample handling for these approaches, as well as limitations of each approach. For example, we observe further loss of non-astrocytes during the wash steps of immunostaining, which may produce additional enrichment. Conversely, low-level expression of GFAP mRNA in non-astrocytes (such as Müller cells) in whole retina controls may be driving underestimates of enrichment by RT-qPCR due to the relative abundance of these cell types. We therefore consider our enrichment estimates—245-fold by quantitative microscopy versus 11.4-fold by RT-qPCR—to represent upper and lower limits on the enrichment produced by the pull-off, and it is likely that a less relative measure of gene expression, such as transcripts-per-million values generated by RNA-sequencing, would clarify this discrepancy. Regardless, even the lower estimate indicates that pull-off microdissection rapidly enriches retinal astrocytes by an order of magnitude without the need for transgenic animals, producing samples suitable for immunostaining, RNA isolation, and potentially other applications. While we only characterize the pull-off as implemented in mice in this study, we initially developed the approach with rat retinas; based on the minimal changes required to adapt the technique to mice, we believe it could be modified to allow for the isolation of retinal astrocytes from other species as well.

Although the pull-off approach on its own can produce astrocytic samples suitable for immunostaining and RNA-based assays, we demonstrate that combining it with the use of transgenic animals and Ribotag immunoprecipitation further enhances its ability to enrich for astrocytic RNA, resulting in total enrichment of at least two orders of magnitude. The selectivity of this approach is supported by additional immunostaining evidence, and it performs well with both naïve retinas and those exposed to elevated IOP via microbead injection, a common ocular hypertension model that recapitulates the loss of retinal ganglion cells in glaucoma. We anticipate that the potent enrichment this technique achieves will aid us in further elucidating the functions of retinal astrocytes and hope that our sharing of this flexible approach with others will enable the development of yet more methods to understand these mysterious cells and other similarly elusive sparse populations.

### Potential pitfalls and trouble shooting

Although highly reproducible once established, the success of the pull-off depends on careful execution and may require slight modifications if in vivo treatments have been performed on the eye. Whether proceeding to subsequent immunoprecipitation or not, we strongly recommend first familiarizing oneself with the microdissection procedure on untreated animals until a degree of familiarity with the technique is acquired. Although subsequent immunoprecipitation is less “technique dependent”, the additional steps and the homogenization of the sample make it a substantially more challenging endpoint to work back from during troubleshooting, whereas microdissected samples can be inspected immediately after isolation.

### Reagents

- Avoid repeated freeze–thaw of ‘Supplemental Reagents’ stored at − 80 °C.
- Collagenase strength varies by lot. We recommend a working concentration between 75 and 150 U/ml (we typically use 100 U/ml). The higher end of this range produces thinner sections with a lower proportion of non-astrocytes, but which are more delicate. Conversely, concentrations at the lower end produce more robust samples with higher numbers of non-astrocytic cells.
- Collagenase activity is pH-sensitive; we recommend a pH of 7–8.
- aPES filter should be used with the ‘matte’ side contacting the photoreceptor layer of the retina. This can be readily distinguished from the glossy finish on the inverse side by examination via dissection microscope, the matte side is covered with small bumps, while the glossy side has diagonal striations.

### Pull-off

- By far the biggest source of difficulty while learning the pull-off technique is non-adherence of tissue to the coverslip, as it makes troubleshooting highly opaque. Tissue damage or over-drying is preferable to non-adherence, as by comparison these are relatively straightforward issues to correct.
- Step 1: Enucleation—Enucleation with blunt forceps is a straightforward option for untreated eyes from adult mice, however additional care may be required for the successful enucleation of eyes treated with ocular hypertension models to prevent collapse or rupture of the globe.
- Steps 3–9: Dissection—We have detailed our own dissection approach, but other groups may have approaches they are already comfortable with. In such cases, the key criteria for successful isolation are removal of the vitreous and flattening of the retina.
- Step 6: Removal of Vitreous—Excess vitreous greatly impacts tissue adherence, leading to failed isolations; as such, removal/minimization of vitreous remaining on the retina is essential. Having tested a mix of approaches for vitreous removal (forceps, ‘jetting’ PBS from a 200 µl pipette, and the use of a soft brush), we have found a clean fine-point watercolor brush (5/0 or 3/0, synthetic sable fur) to be the most reliable for vitreous removal, while minimizing damage to the retina.
- Step 6: Removal of Vitreous—Until familiarity with vitreous removal is achieved, 0.4% trypan blue can be used to visualize the vitreous. It is also initially preferable to risk minor retinal damage due to handling than non-adherence due to excess vitreous, as the former is easier to troubleshoot than the latter.
- Step 15: Incubation—The duration of incubation and volume of collagenase working solution influence not only the extent of enzymatic digest, but also the adherence of tissue to the coverslip. We recommend adjusting enzyme concentration, rather than these factors, during optimization. However, lab conditions such as ambient humidity levels may dictate that some changes must be made; we have found over-dried samples (from longer incubations or smaller volumes of working solution) to be easier to troubleshoot than overly wet samples, which often result in non-adherent tissue.
- Step 17: Lifting the Coverslip—After standardizing the pull-off, the most common source of failure is incomplete adherence of the inner limiting membrane to the coverslip, particularly at the outer edge of the sample. When this occurs, the entire sample can be lost as nonadherent regions anchor the membrane to the retina; however, the tips of the forceps being used to lift the coverslip can be carefully used to sever these connections. This will impact the appearance of the tissue (for IHC) and decrease the amount of tissue isolated, but otherwise will not interfere with downstream applications.

### Immunoprecipitation

- If experiencing difficulty generating adequate RNA yields during immunoprecipitation, multiple pull-off samples can be pooled; use 2 µl of anti-HA antibody and 50 µl of bead suspension per 150 µl of supernatant from the centrifuged lysate.
- Steps 20–26: Homogenization—As with the retinal dissection, there may be room for flexibility with these steps as long as the tissue is adequately homogenized/lysed in Homogenization Buffer and remains cold.
- Step 28: Collection of Input—We strongly recommend taking an input sample, as it aids in determining the relative efficacy of enrichment by the pull-off and immunoprecipitation. It is also an invaluable aid for troubleshooting the immunoprecipitation.
- Step 38: RNA Collection—RNA in RNA Buffer (RLT + β-mercaptoethanol) is quite stable at − 80 °C. This is the preferred stopping point for sample storage, rather than freezing of microdissected tissue.

### Materials and supplies

Items marked with an (*) are required for immunoprecipitation, but not for the pull-off alone.

### Equipment and instruments

The particular model of favored instruments is listed, but equivalents may be substituted.

**Table:** 

Item	Suggested model
Programable heat block (for 37°C incubation)	Any
Stereo Microscope with adj. zoom & ring light	Leica M60
Sonicator (optional, for homogenizing controls)	Fisher Scientific Sonic Dismembrator Model 100
Refrigerated Benchtop Centrifuge	Sorvall Legend Micro 21R
Pipette set & tips (10, 20, 200, 1000 µl)	Any
Blunt Curved Forceps	FST 11152–10
Straight forceps w/ #5 tips	FST 11200–14
Angled forceps	FST 11251–35
Spring scissors	FST 15003–08
Fine-tip watercolor brush	Princeton Aqua Elite 5/0 Round; 4850R-5/0
Tube Rotator*	VWR Mini Tube Rotator
Magnetized Tube Rack*	Invitrogen DynaMag-2

### Critical reagents

**Table:** 

Item	Product information
Lyophilized Type 2 Collagenase (see Reagent Notes)	Worthington, LS004174
HBSS (w/ Ca^2+^, Mg^2+^, Sodium bicarbonate, & Phenol Red)	Sigma, H9269
aPES Filter (See Reagent Notes)	Thermo Fisher Scientific, 595–4520
Round Glass Coverslips (12mm diameter, #1 thickness)	Bellco, 1943-10012A
Mini Trans-Blot Filter Paper	Bio-Rad, 1,703,932
Aluminum Weight (200mg, 5mm × 5 mm)	See Reagent Notes
β-Mercaptoethanol (see Reagent Notes)	Sigma-Aldrich, M3148
Anti-HA.11 Epitope Tag Antibody*	Biolegend, 901,513
Pierce Protein A/G Magnetic Beads*	Thermo Fisher Scientific, 88,803
Potassium Chloride Solution*	Invitrogen, AM9640G
Magnesium Chloride Solution*	Invitrogen, AM9530G
NP-40 Surfact-Amps Detergent Solution*	Thermo Fisher Scientific, 28,324
RNAsin Plus Ribonuclease Inhibitor*	Promega, N261B
Protease Inhibitor Cocktail, General Use* (see Reagent Notes)	VWR, M221-1ML
Dithiothreitol (DTT)* (see Reagent Notes)	VWR, 0281-5G
Cycloheximide* (see Reagent Notes)	Thermo Fisher Scientific, AC357420050
Heparin* (see Reagent Notes)	Thermo Fisher Scientific, BP2524250

### Additional supplies

**Table:** 

Item	Product information
Nuclease-free water	Invitrogen, 10,977,015
1.5ml microtubes	Eppendorf, 022363204
10 × phosphate buffered saline (PBS) w/o Mg^2+^ or Ca^2+^	Thermo Fisher Scientific, J75889.K8
16% Paraformaldehyde	Ted Pella Inc, 18,505
35mm petri dish	Fisher Scientific, FB0875711YZ
100mm petri dish	Fisher Scientific, FB0875712
Kimwipes	Kimberly-Clark, 34,155
#11 Scalpel blades	Bard-Parker, 371,311
25G Needles	Becton Dickinson, 305,125
Tissue Culture Plate, 24 Well, Flat Bottom	Corning, 353,047
Trypan Blue (0.4%) (Optional)	Sigma, T8154
Qiagen RNeasy Plus Micro Kit	Qiagen, 74,034
Takara SMART-Seq HT kit	Takara Bio, 634,437
Agilent RNA 6000 Pico kit	Agilent, 5067–1513

### Antibodies and nuclear counterstains

**Table:** 

Item	Product information
Chicken polyclonal anti-GFAP (1:2000)	Abcam, ab4674
Rabbit monoclonal anti-CRALBP (1:200)	Abcam, ab243664
Mouse monoclonal anti-HA (1:500)	Biolegend, 901,514
Goat polyclonal anti-Iba-1 (1:200)	Novus, NB-100–1028
Alexa Fluor 488-AffiniPure F(ab’)_2_ Fragment Donkey Anti-Chicken IgG (H + L) (1:800)	Jackson ImmunoResearch, 703–546-155
Alexa Fluor 594-AffiniPure Donkey Anti-Mouse IgG (H + L) (1:800)	Jackson ImmunoResearch, 715–546-150
Alexa Fluor 647-AffiniPure Donkey Anti-Rabbit IgG (H + L) (1:800)	Jackson ImmunoResearch, 711–605-152
Alexa Fluor 647-AffiniPure Donkey Anti-Goat IgG (H + L) (1:800)	Jackson ImmunoResearch, 711–605-147
Hoechst 33,342 (Nuclear Counterstain)	Thermo Fisher Scientific, 62,249

### qPCR primers

**Table:** 

Target gene	Sequence
GAPDH	F 5′-GGTTGTCTCCTGCGACTTCAA-3’
	R 5′-CCTGTTGCTGTAGCCGTATTCAT-3’
GFAP	F 5′-ACATCGAGATCGCCACCTACA-3’
	R 5′-GATTTGGTGTCCAGGCTGGTT-3’
CRALBP/RLBP1	F 5′-AGGGTCTTTGTTCACGGAGAT-3’
	R 5′-TGCCACTAGAGCGTTCCTAAA-3’
PAX6	F 5′- CCAACGGTTGTGTGAGTAAAATTC -3’
	R 5′- GCTTTTCGCTAGCGGTTGCGAAGAAC-3’
NEUROD1	F 5′-AAGCCACGGATCAATCTTCTC -3’
	R 5′-GAATAGTGAAACTGACGTGCCT-3’

### Mouse strains

**Table:** 

B6J.129(Cg)-Rpl22tm1.1Psam/SjJ	JAX: 029977, RRID:IMSR_JAX:029977
B6.Cg-Tg (GFAP-Cre) 73.12Mvs/J	JAX: 012886, RRID:IMSR_JAX:012886

### Preparation of reagents

#### Collagenase

Lyophilized Type 2 Collagenase (Worthington, LS004174) was prepared as a stock solution by dissolving 100 mg in HBSS (Sigma, H9269) to a total volume of 500 µl, which can be aliquoted and frozen at − 80 °C. Working solution was prepared immediately prior to tissue isolation by diluting in room temperature HBSS (pH 7–8) to 100 units/ml (varies by lot, approximately 2 µl of stock solution in 1 ml HBSS).

#### aPES filter

Filter material extracted from Nalgene Rapid-Flow 0.2um bottle top filtration kit (Thermo Fisher Scientific, 595–4520). We extract the asymmetric polyethersulfone (aPES) filter material by cutting around the periphery with a razor or scalpel, using care to avoid injury as well as folding or tearing of the filter. 14 × 14 mm squares of filter should be cut as needed and incubated in room temperature distilled water prior to use.

#### Homogenization buffer and wash buffer

These buffers contain additives (‘Supplemental Reagents’) to inhibit protease and RNAse activity, as well as protein translation, and should be prepared and handled with care. RNasin is provided in liquid form and should be kept at − 20 °C without aliquoting or dilution. DTT, Protease inhibitor, Cycloheximide and Heparin stocks should be dissolved in nuclease-free water, aliquoted, and kept at -80°C. Avoid repeated freeze thaws.

Both Homogenization Buffer and Wash Buffer should be prepared fresh immediately before use (on Day 1 or Day 2, respectively). These buffers must be kept on ice after adding supplements. RNA buffer (Qiagen Buffer RLT w/ β-Mercaptoethanol) can be kept at room temperature for 1 month.

**Table:** 

Supplemental reagents	Stock concentration
DTT (VWR # 97,061–340)	1 M
Protease Inhibitor (VWR # 97,063–970)	‘100x’
RNasin (Promega # N2615)	40 u/µl
Cycloheximide (Thermo Scientific # 357,420,050)	5 mg/ml
Heparin (Thermo Scientific # BP2524250)	100 mg/ml

**Table:** 

Homogenization buffer (~ 300 µl/sample)	Per 1ml
Tris (1M, 7.4 pH)	50 µl
KCl (2M)	50 µl
MgCl_2_ (1M)	12 µl
NP-40	10 µl
H_2_0 (DNase/RNase free)	900 µl

**Table:** 

Supplements for HB	Per 1 ml	[Final]
DTT (1M)	1 µl	1mM
Protease Inhibitor (100x)	10 µl	1x
RNasin (40 u/µl)	5 µl	200 u/ml
Cycloheximide (5mg/ml)	20 µl	100 µg/ml
Heparin (100 mg/ml)	10 µl	1 mg/ml

**Table:** 

Wash buffer (~ 500 µl/sample)	Per 1ml
Tris (1M, 7.4 pH)	50 µl
KCl (2M)	50 µl
MgCl_2_ (1M)	12 µl
NP-40	10 µl
H_2_0 (DNase/RNase free)	900 µl

**Table:** 

Supplements for WB	Per 1ml	[Final]
Protease Inhibitor (100x)	5 µl	0.5x
RNasin (40 u/µl)	2.5 µl	100 u/ml
Cycloheximide (5 mg/ml)	20 µl	100 ug/ml

**Table:** 

RNA buffer (350 µl/sample)	Per 1ml
Buffer RLT (Qiagen)	1ml
β-Mercaptoethanol	1 µl

## Data Availability

The datasets used and/or analyzed in the current study are available from the corresponding author on reasonable request.

## Abbreviations

aPES: Asymmetric polyethersulfone
CNS: Central nervous system
HA: Hemagglutinin
IHC: Immunohistochemistry
IOP: Intraocular pressure
MB: Microbead
RIN: RNA integrity number
